# Intelligent gold nanostars for *in vivo* CT imaging and catalase-enhanced synergistic photodynamic & photothermal tumor therapy

**DOI:** 10.7150/thno.33015

**Published:** 2019-07-13

**Authors:** Lin Zhang, Xiao-Quan Yang, Jian-Shuang Wei, Xing Li, Huan Wang, Yuan-Di Zhao

**Affiliations:** 1 Britton Chance Center for Biomedical Photonics at Wuhan National Laboratory for Optoelectronics-Hubei Bioinformatics & Molecular Imaging Key Laboratory, Department of Biomedical Engineering, College of Life Science and Technology, Huazhong University of Science and Technology, Wuhan 430074, Hubei, P. R. China; 2 Key Laboratory of Biomedical Photonics (HUST), Ministry of Education, Huazhong University of Science and Technology, Wuhan 430074, Hubei, P. R. China; 3 Division of Endocrinology, Diabetes and Nutrition University of Maryland, School of Medicine Baltimore, MD 21201, USA

**Keywords:** CT imaging, catalase, hypoxia, photothermal therapy, photodynamic therapy, singlet oxygen

## Abstract

Photodynamic therapy (PDT) is a clinically approved and minimally invasive form of cancer treatment. However, due to hypoxia at the tumor site and phototoxicity to normal tissues, monotherapies using photosensitizers remain suboptimal. This study aimed to develop a highly selective controlled catalase-enhanced synergistic photodynamic and photothermal cancer therapy based on gold nanostars.

**Methods:** Gold nanostars (GNS) with high thermal conversion efficiency were used as the core for photothermal therapy (PTT) and the shell consisted of the photosensitizer Ce6-loaded mesoporous silicon. The shell was modified with catalase (E), which catalyzes the conversion of hydrogen peroxide to oxygen at the tumor site, alleviating hypoxia and increasing the effect of the photodynamic treatment. Finally, a phospholipid derivative with c(RGDyK) was used as the targeting moiety and the nanoparticle-encapsulating material.

**Results:** The nanoprobe exhibited good dispersion, high stability, and high photothermal conversion efficiency (~28%) for PTT as well as a photodynamic "on-off" effect on Ce6 encapsulated in mesoporous channels. The "release" of Ce6 was only triggered under photothermal stimulation *in vivo*. Due to its targeting ability, 72 h after injection of the probe, the tumor site in mice showed an observable CT response. The combined treatment using photothermal therapy (PTT) and catalase-enhanced photo-controlled PDT exerted a superior effect to PTT or PDT monotherapies.

**Conclusion:** Our findings demonstrate that the use of this intelligent nanoprobe for CT-targeted image-guided treatment of tumors with integrated photothermal therapy (PTT) and catalase-enhanced controlled photodynamic therapy (PDT) may provide a novel approach for cancer theranostics.

## Introduction

In recent years, photodynamic therapy (PDT) has been proposed as a useful tool in oncology 1. In PDT treatment, photosensitizers (PS) can kill nearby cells at specific wavelengths of light. This is because the light causes the PS to transmit absorbed photonic energy to oxygen molecules converting them to reactive oxygen species (ROS) toxic to cancer cells or other targeted cells. This process of killing cells using both light and chemicals to create cytotoxic ROS is referred to as phototoxicity 2, 3.

At present, several problems need to be resolved in PDT. These include insufficient depth penetration by light, skin phototoxicity caused by the non-specific distribution of PS, and the reduced photodynamic effect caused by hypoxia at the tumor site. The excitation wavelengths of traditional PS are usually in the ultraviolet or visible bands and photons of these wavelengths can only achieve shallow penetration and are easily disturbed in tissues by endogenous substances. For example, chromoplastids (e.g. hemoglobin) in most tissues strongly absorb visible light and interfere with the conversion of PS to light energy 4, resulting in inefficient treatment. Therefore, visible light cannot be used for PDT of deep tumors. Near-infrared (NIR, 700 - 1300 nm) has become the preferred light source for PDT due to its increased depth of penetration (10 - 15 mm) 5.

In photodynamic reactions, high quantum yield and suitable wavelength of selected PS are the primary considerations. At present, most of the commonly-used PS contains hydrophobic structures, which tend to self-aggregate in aqueous solution and affect their bioavailability and light absorption *in vivo*, thus limiting their application in PDT. Recent studies demonstrated that appropriate drug delivery systems (such as liposomes 6, polymeric particles 7, and micelles 8) could facilitate the intravenous administration of PS and promote their enrichment in target tissues and prevent their auto-aggregation in blood 9 while simultaneously improving their phototherapeutic activity. However, it has been reported that although nano-formulations can increase the accumulation of PS at tumor sites via the enhanced permeability and retention (EPR) effect, their phototoxicity can still affect neighboring cells. The temporally limited existence of singlet oxygen with limited diffusion distance 10 and poor tumor selectivity can lead to photodynamic damage to neighboring normal cells 11. Chlorin e6 (Ce6) is a second-generation photosensitizer with antitumor activity when used in conjunction with irradiation. Its excitation wavelength of 660 nm penetrates deeper into the tissue, its absorption coefficient is 10 times higher than that of hematoporphyrin, and it demonstrates a high quantum yield of singlet oxygen 12, 13. However, its low selectivity toward cancer tissues inevitably results in damage to normal tissues, particularly the skin. Under sunlight, its phototoxicity leads to skin redness, edema, pain, sunburn, and even ulcers. Therefore, if the photodynamic effect of Ce6 could be controlled and inactivated in normal tissues during the "off" state and activated only at the tumor or lesion site during the "on" state, the damage to healthy tissues can be minimized.

Generally, oxygen supply at the tumor site is insufficient due to incomplete blood vessel growth. The resulting hypoxic condition is not conducive to PDT, as PDT rapidly consumes oxygen at the tumor site in response to irradiation 14-16. Blockage of the tumor blood vessels and local oxygen consumption during PDT can aggravate hypoxia at the tumor site. Therefore, alleviating hypoxia is an important consideration by which the overall photodynamic effect can be improved. Under normal physiological conditions, H_2_O_2_ levels in organisms remain at a low equilibrium level. However, cancer cells produce H_2_O_2_ excessively, which leads to higher levels of H_2_O_2_ in cancer tissues than in normal tissues 17. Based on this observation, researchers have proposed that a catalytic material could be used to induce the decomposition of H_2_O_2_ to produce O_2_, which can then overcome the hypoxic tumor microenvironment. Examples of catalytic materials for the photodynamic hypoxia resistance 18-20 include MnO_2_ 19 and the metal platinum nanoparticles 21-24.

Based on the above limitations of PDT and the shortcomings of Ce6, we designed a novel integrated diagnostic and therapeutic probe. Gold nanostar (GNS) was used as the core and Ce6-loaded mesoporous silicon was used as the shell. The particles were then modified with catalase and the targeting moiety c(RGDyK), which can specifically recognize cancer cells 25,26. Using this composite nanoprobe, we performed a combination of active targeting by photothermal therapy (PTT) and PDT on tumor-bearing nude-mice under the guidance of computed tomography (CT) imaging.

Our probe design has several advantages. First, the atomic number of gold and its X-ray attenuation coefficient are high (5.16 cm^2^/g, 100 eV) 27 thus meeting the requirement for CT imaging. Second, the plasma resonance peak of GNS is 600 - 1000 nm and its corresponding penetration depth is 10 - 15 mm. GNS also has high photothermal conversion efficiency, and after irradiation with a 808 nm laser, a large amount of heat can be produced to kill tumor cells. Third, GNS plays the role of a "switch" in PDT. The high loading efficiency of mesoporous silicon (mSiO_2_) alleviates the solubility problem of Ce6, improves its stability, and prevents toxicity caused by premature leakage of Ce6. Also, Ce6 is encapsulated in mSiO_2_ near GNS, which “turns off ” Ce6, releasing it when the probe reaches the tumor site and when the photothermal effect of GNS is then exerted. This results in the production of a large quantity of singlet oxygen that can kill neighboring tumor cells avoiding untargeted phototoxicity during transport *in vivo*. Fourth, catalase immobilized on the surface of mSiO_2_ can block the mesoporous channels and partially prevent the leakage of PS molecules during transport. Furthermore, when the probe reaches the tumor site, catalase can convert high endogenous concentrations of H_2_O_2_ to oxygen. The presence of excess oxygen solves the problem of tumor hypoxic resistance caused by the oxygen consumption of PDT and improving the effect of PDT. Finally, the targeting peptide c(RGDyK) is connected to the surface of the probe via PEG-phospholipids, which both improves the selectivity of the probe for tumor cells and prolongs the circulation duration of the probe *in vivo*. This promotes the active targeting of the probe to the tumor site in circulation.

Both *in vivo* and *in vitro* experiments showed that our nanoprobe effectively and locally generated high heat to achieve PTT and released Ce6 under 808 nm laser irradiation after targeted binding to the tumor cell. The combined presence of Ce6 and oxygen produced from the catalysis of H_2_O_2_ by catalase produced a large amount of ROS under irradiation with a 660 nm laser. The final results revealed that our probe achieved effective mouse tumor cytotoxicity through synergistic PTT and PDT. This study offers an important reference for the exploration of new photodynamic probes.

## Methods

### Synthesis of GNS

The synthesis of GNS was performed according to the literature with some modifications 28. Briefly, 15 mL of 1% citric acid solution was added to 100 mL of 1 mM boiling chloroauric acid solution, stirred vigorously for 15 min, then cooled to room temperature and stored at 4 ºC until subsequent steps. The solution was added to 100 mL chloroauric acid (0.25 mM), followed by 100 mL hydrochloric acid (1 M), 400 mL silver nitrate (10 mM), and 200 mL ascorbic acid (100 mM). After 30 s of stirring, the solution turned from bright red to blue-black indicating the synthesis of GNS, which was stored at 4 ºC until further use.

### Preparation of mSiO_2_-coated GNS particles loaded with Ce6 (Au@mSiO_2_/Ce6, ASC)

The Au@mSiO_2_ (AS) probe was synthesized using the gel-sol method 29. One mL of hexadecyl trimethyl ammonium bromide (CTAB, 0.1 M) was added to 100 mL GNS synthesized above and stirred for 30 min. Next, sodium hydroxide was used to adjust the pH to 10 and 36 μL tetraethylorthosilane (TEOS) was dripped in at 30 min intervals three times. The mixture was centrifuged at 9600 rpm for 20 min after stirring overnight at 26 ºC, washed with ethanol and water three times, and dispersed into 50 mL anhydrous ethanol. After adding 150 mg ammonium nitrate, the anhydrous ethanol solution was refluxed at 78 ºC for 4 h. The AS dispersion system was obtained after ethanol washing and 100 μL 3-aminopropyltriethoxysilane (APTS) was added to 40 mL AS dispersion. After 48 h of stirring, the amino-modified Au@mSiO_2_-NH_2_ (ASN) particles were obtained following centrifugation and ethanol wash.

The Ce6 photosensitizer was dissolved in DMSO (1 mg/mL) and 100, 200, and 500 μL of Ce6 were added to 10 mL ASN dispersion. Following 48 h of stirring, ASC was obtained after washing with water until the supernatant had no absorption peak remaining at 405 nm.

### Preparation of DSPE-PEG-RGD and catalase co-encapsulated ASC (Au@mSiO_2_/Ce6@Catalase@DSPE-PEG-RGD, ASCE-R)

Four mg catalase and 4 mg EDC·HCl were added to 10 mL ASC dispersion and stirred for 24 h at room temperature, Subsequently, the excess catalase and EDC·HCl were removed by centrifugation at 12,000 rpm for 10 min and Au@mSiO_2_/Ce6@Catalase (ASCE) was obtained which was dispersed in 5 mL water. Next, 2 mg of DSPE-mPEG (D) and 1 mg of DSPE-PEG-RGD (R) were added to the ASCE dispersion. After 3 h of ultrasonication, the dispersion was centrifuged at 12,000 rpm for 10 min and washed three times to remove any excess phospholipid derivatives yielding ASCE-R.

### Preparation of multiple probes

Two mg D, 1 mg R (please refer to the abbreviations in Table 1), and 3 mg Ce6 were added to 3 mL chloroform with sonication for 15 min. The mixture was then stirred and slowly dried under vacuum pump. When chloroform was completely evaporated, the solids were dispersed in 1 mL ultrapure water, transferred to an ultrafiltration tube (MWCO=100 kDa) to further remove excess phospholipid molecules, and finally dispersed in PBS to obtain the Ce6@DSPE-PEG-RGD (C-R) probe. The filtrate was collected after centrifugation and its absorption at 405 nm was determined. The loading rate of Ce6 was calculated according to the standard curve of Ce6 concentration and absorption value. The formula of the loading rate was as follows:

loading rate (%) = (amount of loaded Ce6 - amount of lost Ce6)/amount of the freeze-dried probe

Contrast probes such as Au@mSiO_2_@DSPE-PEG (AS-D), Au@mSiO_2_@DSPE-PEG-RGD (AS-R), Au@mSiO_2_/Ce6@DSPE-PEG-RGD (ASC-R) were obtained using the above protocol and their respective starting materials (Table 1).

### Probe stability test

Nine portions of freeze-dried ASCE-R samples were dissolved in ultrapure water, DMEM, or DMEM+10% serum and stored at 4, 25, and 37 ºC. Particle size, polydispersity index (PDI), and zeta potential were determined during storage. The stability of the probe was tested every 7 d.

### CT response of probe and CT imaging in cells and *in vivo*

ASCE-R at different concentrations (1.07, 5.47, 7.86, 11.17, 15.72, and 34.4 mg/mL) was used to investigate the CT response on CT imaging system.

HeLa and MCF-7 cells in logarithmic growth phase were inoculated in 6-well plates. After incubation in a 5% CO_2_ incubator for 24 h, their media were removed and replaced by serum-free media containing the same concentrations of ASCE-R and ASCE-D (108 μg/mL). In control groups, the cells were cultured without probes. After incubation for 6 h, cells were collected and fixed with 2.5% glutaraldehyde for 30 min for CT imaging.

HeLa cells were subcutaneously inoculated in 4-week-old BALB/c male nude mice (SPF grade). When the tumor volume reached 50 - 120 mm^3^, 200 μL ASCE-R (5 mg/mL) or ASCE-D (5 mg/mL) probes were injected into the tail vein of the mice for CT imaging at different times. The nude mice were anesthetized by respiratory anesthesia system and fixed on the imaging platform. All animal experiments were approved by the Ethics Committee of Animal Laboratory of Huazhong University of Science and Technology.

### Probe photothermal stability and conversion efficiency

The 300 μL ASCE-R probe (108 μg/mL) was irradiated using 808 nm laser (0.5, 1.0, 1.5, and 2.5 W/cm^2^) for 5 min and the temperature change in the sample was recorded using an infrared thermal imager. Different concentrations of ASCE-R probe (0, 54, 108, and 1080 μg/mL) were tested for sample temperature change after irradiation with the same laser (1.0 W/cm^2^). Each group had four parallel settings.

After measuring the absorption value of the ASCE-R probe at 808 nm, 0.2 mL of the probe was placed in a quartz colorimetric dish and irradiated with an 808 nm laser (2.5 W/cm^2^) for 5 min. The temperature change of the entire system was recorded after the laser was turned off. The photothermal conversion efficiency of the probe was calculated by 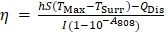
according to a previously published reference 30. In the equation, h is the heat transfer coefficient, *S* is the surface area of the container, *T*_max_ is the highest temperature after illumination, *T*_surr_ is the initial temperature upon illumination, *Q*_Dis_ is the energy emitted by the solute, *I* is the laser intensity, and *A*_808_ is the absorbance value of the probe at 808 nm.

### Fluorescence detection of multiple probes

The fluorescence emission spectra of ASCE-R (108 μg/mL), Ce6 (2.5 μg/mL), ASE-R (108 μg/mL), GNS (108 μg/mL), and AS (108 μg/mL) were measured. After irradiating the ASCE-R probe with 808 nm laser (2 W/cm^2^) for 0, 1, 3, 5, 8, and 15 min, the fluorescence spectrum of the supernatant was evaluated following centrifugation to determine the release of Ce6.

### Measurement of oxygen production capacity of the probe and its effect on singlet oxygen production with laser irradiation

Different concentrations of ASCE-R (0, 60, 120, 240, 480, and 960 μg/mL) were added to H_2_O_2_ (1 mM) and the oxygen production capacity of the probe was measured by portable dissolved oxygen meter over 2 min. At the same concentration, the oxygen production capacity of the ASE-R, ASB-R, and ASC-R probes was also determined.

The absorption of 1, 3-diphenylisobenzofuran (DPBF) solution (DPBF/DMSO, 10 mM) was measured at 415 nm at different times under irradiation with 808 nm (1.0 W/cm^2^), 660 nm (0.1 W/cm^2^), 808/660 nm (successively irradiated with the 808 nm laser (1.0 W/cm^2^), and the 660 nm laser (0.1 W/cm^2^) unless otherwise described.

### Probe cytotoxicity

HeLa and MCF-7 cells in logarithmic growth phase were seeded in 96-well plates. After 24 h incubation in a 5% CO_2_ incubator at 37 ºC, the media were removed. Serum-free media containing different concentrations of ASC-R (0, 36, 43, 72, 108, and 216 μg/mL) were added to the cells. Six parallel wells were set up for each group. After 24 h, the media were removed and cells were washed three times with PBS. The fresh serum-free medium was added with 20 μL thiazole blue (5 mg/mL) to each well. After incubation for 4 h, the media were removed. A 150 μL volume of DMSO was added to each well and the culture plate were placed on a shaker for 20 min. The cell survival rate was calculated by measuring the absorption at 490 nm with Elx-808 enzyme marker.

HeLa cells were cultured under the same conditions. Serum-free media containing different concentrations of ASCE-R (0, 36, 43, 72, 108, and 216 μg/mL) were added to HeLa cells. Cell survival rate was calculated as described above.

### Probe targeting labeling

HeLa and MCF-7 cells were cultured under the same conditions and the same concentration of ASCE-R (108 μg/mL) was added to both cell types. After 6 h, the cells were fixed with 2.5% glutaraldehyde. After 48 h, the cells were rinsed with 0.1 M PBS (pH = 7.0) three times and fixed at room temperature for 4 h with PBS (pH = 7.4) containing 1% osmium acid. The samples were then rinsed with PBS (pH = 7.4) 3 times for 15 min each time. Gradient dehydration using 0, 80, 85, 90, 95, and 100% alcohol for 15 - 20 min was carried out twice. After 8 - 12h of treatment with different proportions of penetrants (2:1 and 1:1) consisting of acetone and epoxy resin, ultra-thin sections of 80 - 100 nm were prepared with an EM UC7 microtome (Leica, Germany) and embedded in epoxy resin at 60 ºC for 48 h. Cell morphology was observed by transmission electron microscopy (TEM).

After HeLa cells were cultured under the same conditions, serum-free medium containing ASC-R (36 μg/mL) was added. After incubation for 0, 2, 4, 8, 12, and 24 h, 50,000 cells were quantified by flow cytometry. The fluorescence intensity of Ce6 was analyzed to investigate the nternalization of probes in HeLa cells.

### Photothermal, phototoxic, and photodynamic tests of the probe

HeLa cells were cultured in a 5% CO_2_ incubator at 37 °C for 12 h followed by the addition of 100 μL serum-free medium containing ASC-R (0.8, 2, and 4 μg/mL Ce6) and C-R (0.8 and 2 μg/mL Ce6). After 4 h, PBS was removed and cells were washed three times. Cells from each group were divided into three groups and irradiated with 808 nm laser (1.0 W/cm^2^) for 3 min, 660 nm laser (0.1 W/cm^2^) for 8 min, or 808/660 nm laser (3 min with 808 nm laser and 8 min with 660 nm laser). After incubation in darkness for 24 h, 20 μL thiazole blue (5 mg/mL) was added and allowed to incubate for 4 h. The cell survival rate was calculated by measuring absorption at 490 nm.

To investigate the phototoxicity of the particles, 100 μL serum-free medium containing Ce6 (2 and 4 μg/mL) was added to HeLa cells under the same conditions. Four hours later, cells were removed and washed with PBS three times. Fresh medium was added to HeLa cells, which were exposed to a fluorescent lamp for 6 h. After 24 h, thiazole blue (5 mg/mL) was added for 4 h and the cell survival rate was calculated by measuring the absorption at 490 nm.

HeLa and MCF-7 cells were cultured under the same conditions. A 500 μL volume of serum-free media containing ASD (38 μg/mL) and AS-R (38 μg/mL) was added. After 4 h, the media were removed, cells were washed three times with PBS, and irradiated with 808 nm laser (1 W/cm^2^) for 5 min. Subsequently, the cells were stained with calcein (AM) and propidium iodide (PI) for 5 min, the staining solution was removed washed three times with PBS, and the stained cells were observed under a fluorescence microscope.

HeLa cells were cultured under the same conditions. The cells were cultured in 500 μL serum-free medium containing ASC-R (38 μg/mL, approximately 1 μg/mL Ce6). After 4 h, the medium was removed and cells were washed three times with PBS. Then, the cells were divided into three groups. The first group was irradiated with 808 nm laser (1.0 W/cm^2^) for 5 min; the second group was irradiated with 660 nm laser (0.1 W/cm^2^) for 8 min, and the last one with 808/660nm laser (808 nm with 5 min and 660 nm with 8 min). After 5 min of PI staining, the dye solution was removed and cells were washed three times with PBS and observed under a fluorescence microscope. A 500 μL volume of C-R (1 μg/mL Ce6) was added to HeLa cells and the cells were irradiated with a 660 nm laser (0.1 W/cm^2^) for 8 min as control.

HeLa cells were cultured under the same conditions and 500 μL serum-free media containing ASC-R, ASCE-R (38 μg/mL, approximately 1 μg/mL Ce6), or C-R (1 μg/mL Ce6) were added. After 4 h, the media were removed and cells were washed with PBS three times. Then, 500 μL serum-free medium containing 2', 7'-dichlorodihydrofluorescein diacetate (DCFH-DA, 10 μM) was added. After irradiation with the 808 nm laser (1 W/cm^2^) for 10 min, cells were cultured for 0.5 h, washed three times with PBS to remove free DCFH-DA, and fixed with 2.5% glutaraldehyde for 30 min followed by observation under a fluorescence microscope.

### Anti-tumor effect *in vivo*

The ASCE-R (8 mg/mL, approximately 0.2 mg/mL Ce6), ASC-R (8 mg/mL, approximately 0.2 mg/mL Ce6), and C-R (0.2 mg/mL Ce6) probes were injected into the tumors of HeLa-inoculated BALB/c nude mice. After 4 h, a laser was used to irradiate the mice and the mice were sacrificed after 1 h at ambient temperature. The spleen, lung, kidney, small intestine, and tumor were removed and placed into centrifuge tubes, repeatedly cleaned, then immersed in 4% paraformaldehyde. After 48 h of immersion, sections were made and stained with hematoxylin-eosin (HE) stain, and hypoxic areas were stained with DAPI (blue), anti-CD31 antibody (red), and HIF-1 antibody (green). The sections were observed by microscopy.

Forty-five BALB/c nude mice inoculated with HeLa tumors and were divided into 9 groups. The mice were injected with 150 μL PBS, ASCE-R, ASC-R, or C-R into the tumor for 4 h. The ASCE-R(I) and the ASC-R(I) groups were irradiated with the 808 nm laser followed by the 660 nm laser; the ASCE-R(II) and the ASC-R(II) groups were irradiated with the 808 nm and 660 nm laser only, respectively; the C-R(I) group was only irradiated with the 660 nm laser, while the C-R(II), ASCE-R(III), and PBS groups were not irradiated. The final group was irradiated with 808/660 nm without probe injection and was named the 808/660 nm group. The weight and tumor volume of the mice were recorded by digital vernier caliper and body weight meter every 2 d, and the animals were photographed every 4 d. During the irradiation period, the duration of irradiation was 10 min, the power of the 808 nm laser was 2 W/cm^2^, and the power of the 660 nm laser was 0.2 W/cm^2^, with thermal imaging as output.

### Biocompatibility of probe *in vivo*

Fifty-four-week-old BALB/c male mice (SPF grade) were randomly divided into two groups with 25 mice in each group. One group was injected with 150 μL ASCE-R (8 mg/mL) into the tumor and the other group was injected with 150 μL PBS as a control. Blood samples were collected from the animals' eyeballs on days 0, 1, 3, 7, and 15. A 200 μL volume of fresh blood was taken for liver enzyme analysis (aspartate aminotransferase (AST), alanine aminotransferase (ALT)). Additionally, 100 μL blood was collected for blood analysis (red blood cell (RBC), white blood cell (WBC), platelet (PLT) and hemoglobin (HGB)).

## Results and Discussion

### Characterization of probes

**Figure 1:**
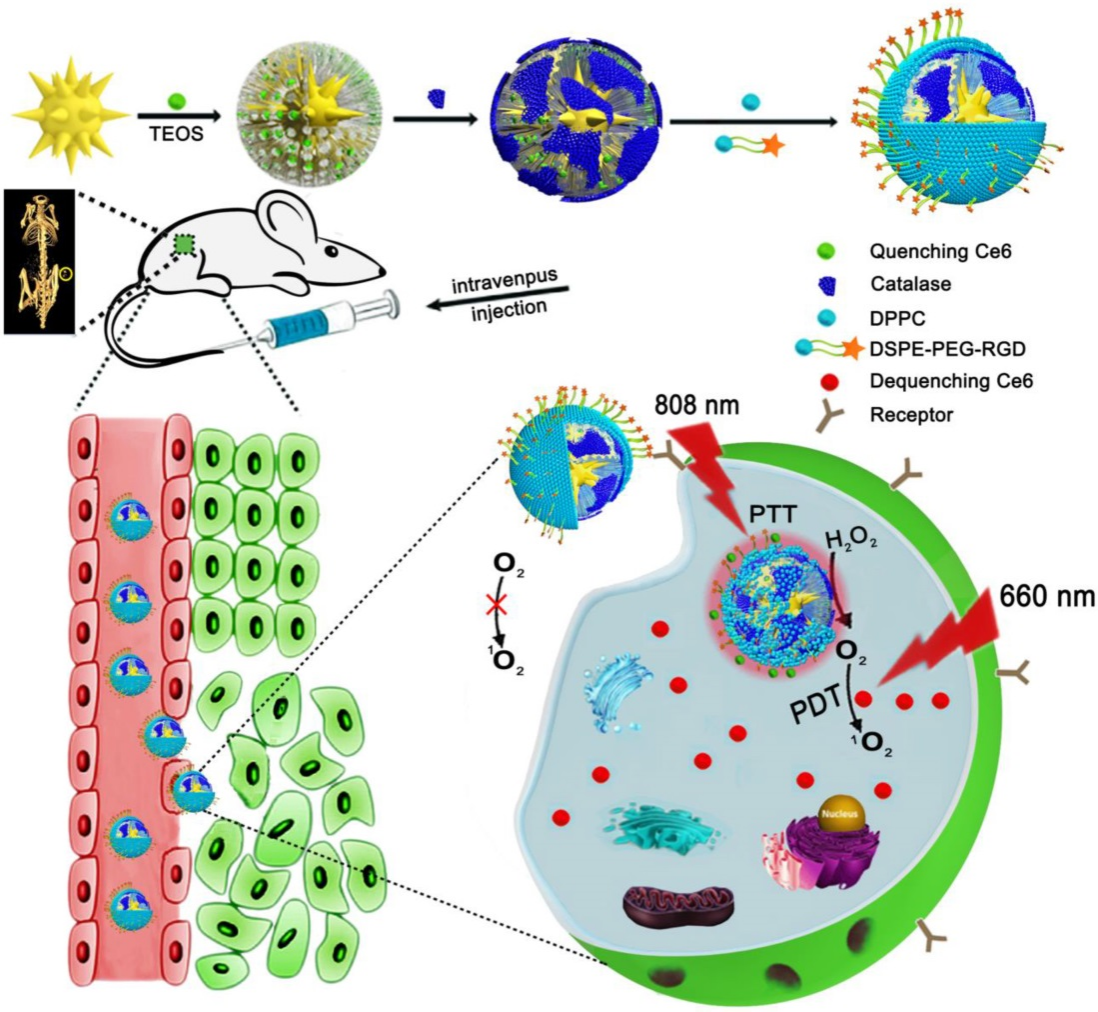
The preparation process of the ASCE-R probe and schematic diagram of tumor-targeted imaging and therapy.

This study designed a spherical multifunctional nanoprobe (ASCE-R) using phospholipid self-assembly, c(RGDyK), which binds integrin α_v_β_3_ receptors on tumor cells, as the target recognition molecule, GNS as the CT contrast agent, and the porphyrin-based small molecule Ce6 loaded in mesoporous silica channels as the photosensitizer. HeLa cells with high expression of integrin α_v_β_3_ receptor were used as the tumor model. The nanoprobe was administered in tumor-bearing nude mice for targeted CT imaging, PTT, and PDT (Figure 1). After arriving at the tumor site, the probe was internalized into the tumor cell by the recognition receptor.

The probe we designed has many advantageous features: Phospholipid R is a dense colloidal crystal at normal body temperature preventing water and the embedded drug to diffuse out. Under laser irradiation at 808 nm, the nanoparticle-generated heat rapidly increases the surrounding temperature beyond the phase transition temperature (41 °C) of R. The phospholipid membrane transited from a gel-like state to a liquid crystal state increasing the fluidity of the membrane making it permeable to water or the drug 31-33. Simultaneously, the acidic environment in the tumor cell micromilieu accelerates the hydrolysis of phospholipids 34, 35 and the large quantity of hydrogen peroxide oxidizes unsaturated acyl chains in the phospholipid molecule 36 further enhancing the fluidity of the phospholipid layer. These factors lead to the breakage of the phospholipid membrane. Under the concurrent laser irradiation, GNS produces a large amount of thermal energy 37-39 destroying the weak electrostatic interaction between the carboxyl group of Ce6 and the amino group 40-42 on the inner surface of the channel 43, 44 thereby releasing Ce6 from the channel. In the acidic tumor microenvironment, protonation of the carboxylic group in Ce6 also weakens its electrostatic interaction with the amide on the inner surface of the pore, also assisting in the release of Ce6 from the pore 45, 46. Following irradiation by the 660 nm laser, Ce6 transmits absorbed photonic energy to oxygen molecules and a large amount of singlet oxygen with oxidative activity is produced 47-49.

**Figure 2:**
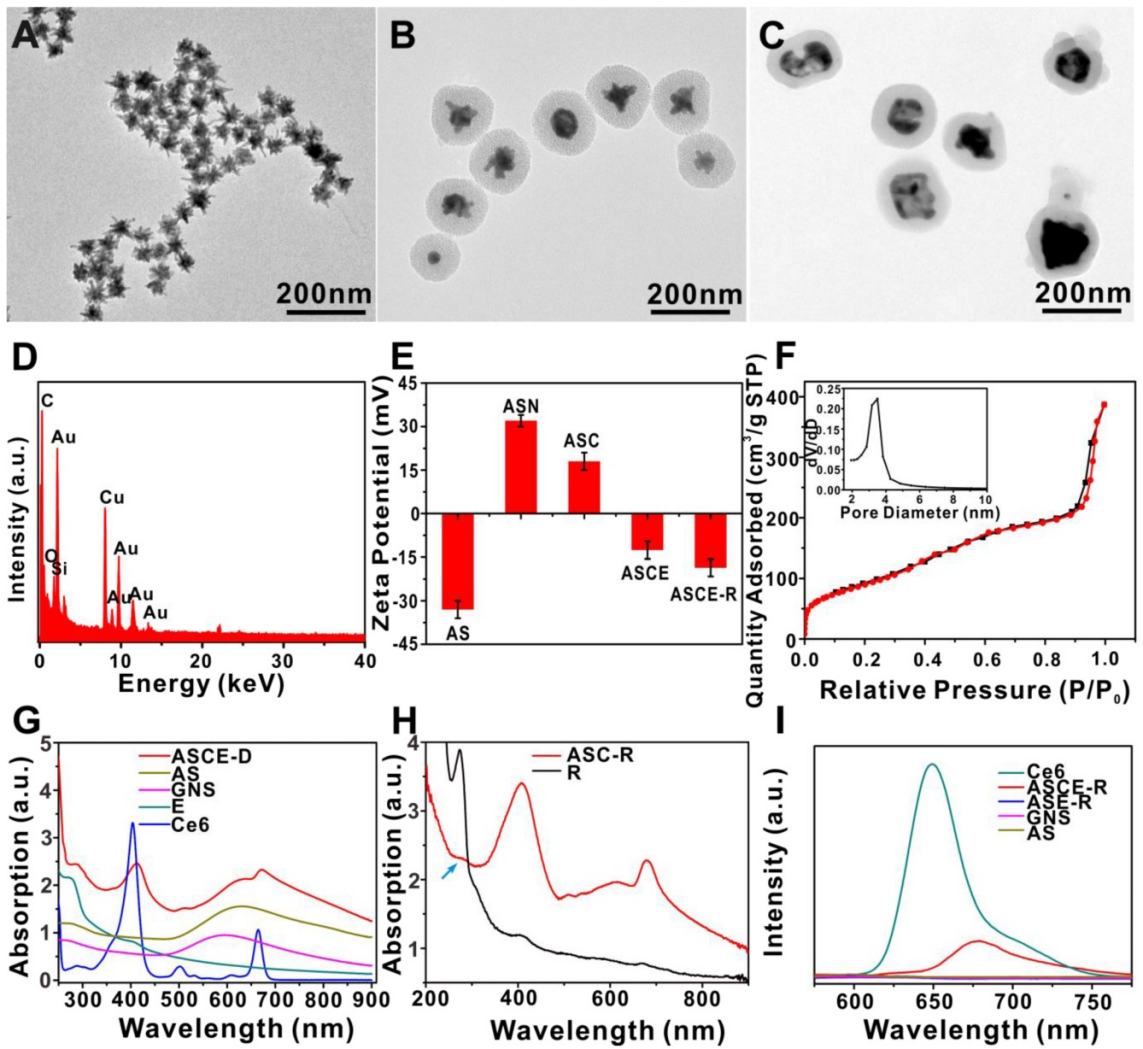
TEM of GNS (A), AS (B) and ASCE-R (C); EDS of ASCE-R (D); zeta potentials of different probes (E); N_2_ adsorption-desorption isotherm curve and pore size distribution curve of AS (illustration) (F); ultraviolet-visible absorption spectra of different probes (G); ultraviolet-visible absorption spectra of R and ASC-R (H); fluorescence spectra of different probes (I).

The probe was then characterized using different methods. TEM showed that the diameter of GNS ranged from 60 to 100 nm and contained many "spiny" structures. The thickness of the mSiO_2_ layer was about 60 to 80 nm, and the pore channels were visible. After coating with catalase and phospholipids, the nanoparticle size did not change significantly, but the pore channels became indistinguishable (Figure 2A - C).

Energy dispersive spectrometer (EDS) showed that the probe contained gold, oxygen, and silicon (Figure 2D). The mSiO_2_-coated GNS had negative zeta potential (-33 ± 5 mV) and the zeta potential of amino-modified ASN changed to 32 ± 2.1 mV due to the positive charge of the NH_2_ group. After Ce6 loading and catalase modification, the value decreased to -12.6 ± 3.3 mV and following phospholipid encapsulation, the final zeta potential value of the ASCE-R probe was -18.2 ± 5.6 mV (Figure 2E). These changes were consistent with the gradually introduced alterations of the material. To load Ce6 small molecule effectively, the mesoporous channels were examined. Nitrogen adsorption-desorption isotherms of AS showed an average pore size of 5.7 nm and specific surface area of 333.67 m^2^/g, indicating that the probe had a large adsorption capacity and could meet the loading requirement of Ce6 (Figure 2F).

The absorption spectrum of the probe was also studied and showed a red-shift of about 30 nm from 600 nm in the GNS absorption peak when it was mSiO_2_-coated. After Ce6 loading, enzyme modification, and phospholipid coating, four strong absorption peaks appeared at 278, 405, 630, and 660 nm for ASCE-D belonging to the characteristic absorption peaks of catalase, Ce6, GNS, and Ce6, respectively, indicating that they were successfully coated onto the nanoprobe (Figure 2G). Since the characteristic absorption peak of the short peptide linked to the phospholipid overlaps with that of catalase at 275 nm (blue arrow in Figure 2H), it was difficult to judge whether the particle surface was modified with the targeting peptide c(RGDyK). Therefore, ASC-R probe without catalase was prepared using the same procedure. Results showed an absorption peak at 275 nm, indicating that the short peptide was successfully linked to the probe (Figure 2H).

The fluorescence peak of Ce6 exhibited a red shift from 650 nm to 680 nm when it became encapsulated in the probe channels in ASCE-R. However, its fluorescence intensity decreased significantly even at the same concentration, indicating that the GNS core had a significant quenching effect on Ce6 loaded into mSiO_2_ keeping Ce6 in an "off" state and inhibiting its luminescence. Since ASE-R, GNS, and AS did not contain Ce6, no fluorescence peak was observed in these probes (Figure 2I).

The AS and AS-R probes were prepared using the same procedure. The IR spectra indicated that AS-R contained C-H bond asymmetry (2924 cm^-1^), symmetrical stretching vibration peak (2853 cm^-1^), N-H single substitution (1649 cm^-1^), and double substitution stretching vibration peak (1563 cm^-1^), which were consistent with the presence of R. Both AS and AS-R showed Si-O bond stretching vibration peak (1092 cm^-1^), suggesting that the phospholipid derivative, which contained the short peptide, c(RGDyK) successfully enclosed the surface of the probe (Figure S1).

The investigation of the stability of the probe showed that the particle size remained stable with time (Figure S2A - C). Furthermore, the PDI (Figure S2D - F) and the zeta potential (Figure S2H - J) changed slightly in different media at different temperatures indicating good stability of the multifunctional composite probe. We also examined the stability of ASCE-R in different physiological environments of water, HEPES, PBS (pH = 7.4), and bovine serum albumin for seven days. ASCE-R showed no aggregation and precipitation and the colloidal dispersion system was stable for 7 d. The particle size, PDI and zeta potential of the particles in PBS (pH=7.4) at 25 and 37 °C are shown in Figure S3.

### CT imaging of probes *in vitro* and* in vivo*

**Figure 3:**
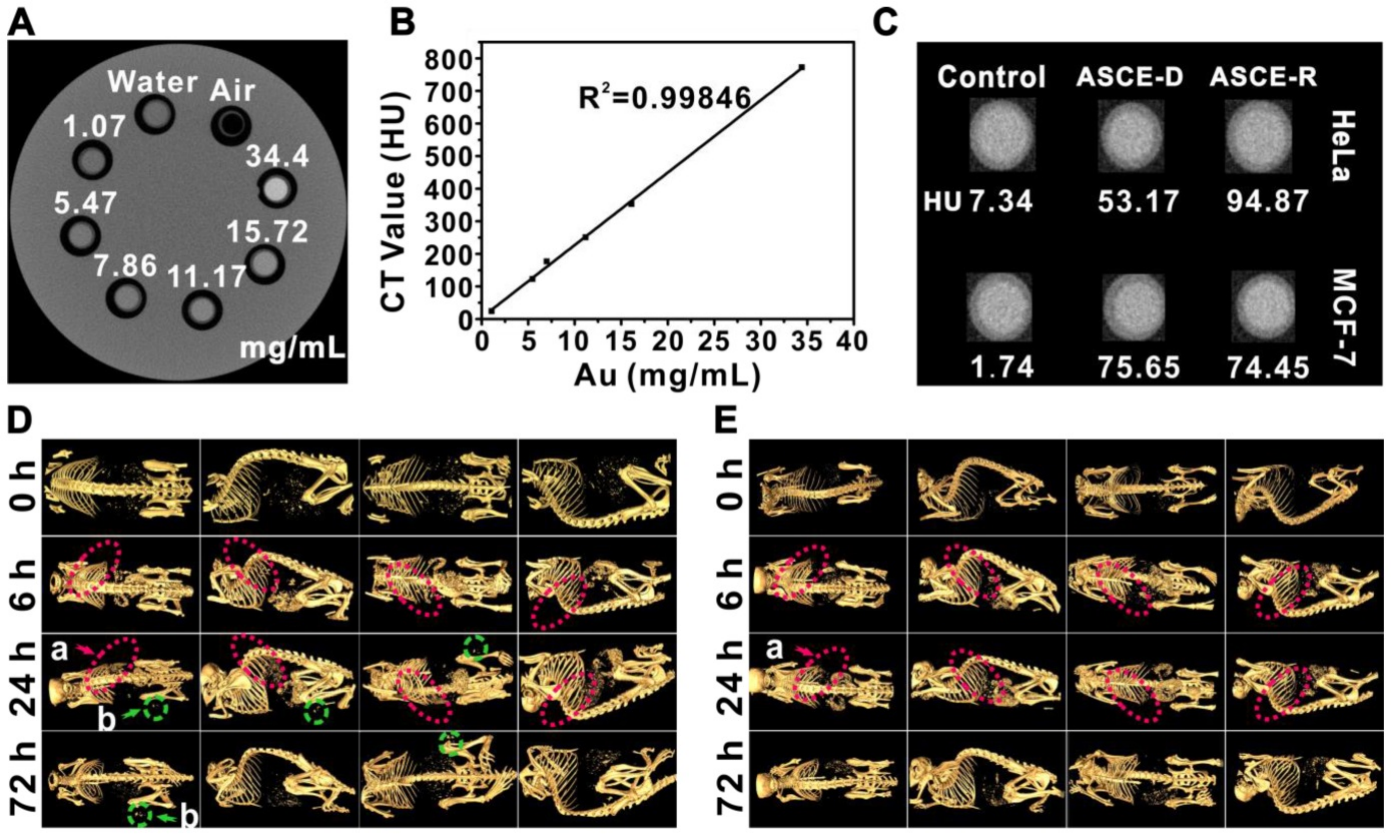
CT imaging (A) of ASCE-R with different concentrations and the HU value curve of concentration-dependence (B); CT imaging of targeted recognition of receptor-positive HeLa and receptor-negative MCF-7 tumor cells with 108 µg/mL probe (C); CT imaging at different times of nude mice after injection of the ASCE-R positive probe (D) and ASCE-D negative probe (E), a: spleen (marked in red), b: tumor (marked in green).

With increasing probe concentration, CT value of the probe increased gradually, and showed a good linear correlation with the gold concentration in the probe (Figures 3A, 3B). HeLa cells were used as the positive control tumor model cell type due to their high expression of the α_v_β_3_ receptor while MCF-7 cells were used as the negative control due to their low expression of the α_v_β_3_ receptor. After incubation with different probes, CT imaging showed that HeLa cells incubated with ASCE-R, containing the short peptide c(RGDyK), achieved the highest HU value when compared with other groups, indicating that the probe ASCE-R presents outstanding tumor-targeting CT imaging ability *in vitro* (Figure 3C).

The positive probe ASCE-R and negative probe ASCE-D without the short peptide were injected into tumor-bearing nude mice via the tail vein. After 6 h, the spleen of mice injected with the positive probe showed a clear CT signal response compared with pre-injection (Figure 3D), indicating that the probe reached the spleen via circulation. After 24 h, the probe's signal appeared at the tumor site and started clearing from the spleen indicating that probe enrichment had begun in the tumor. After 72 h, the CT signal of spleen gradually disappeared, and the tumor site became more apparent compared with the CT scan taken at 24 h. However, in the negative control group, the probe appeared after 6 h in the spleen where the CT signal reached its maximum at 24 h, then began to attenuate. In contrast, no significant CT signal was observed at the tumor site for the duration of the experiment when compared with pre-injection (Figure 3E). These experiments demonstrated that following ASCE-R probe injection, the probe with c(RGDyK) peptide can accurately target the HeLa tumor with high expression of integrin α_v_β_3_ receptor.

### Photothermal effect and O_2_ generation by probes

**Figure 4:**
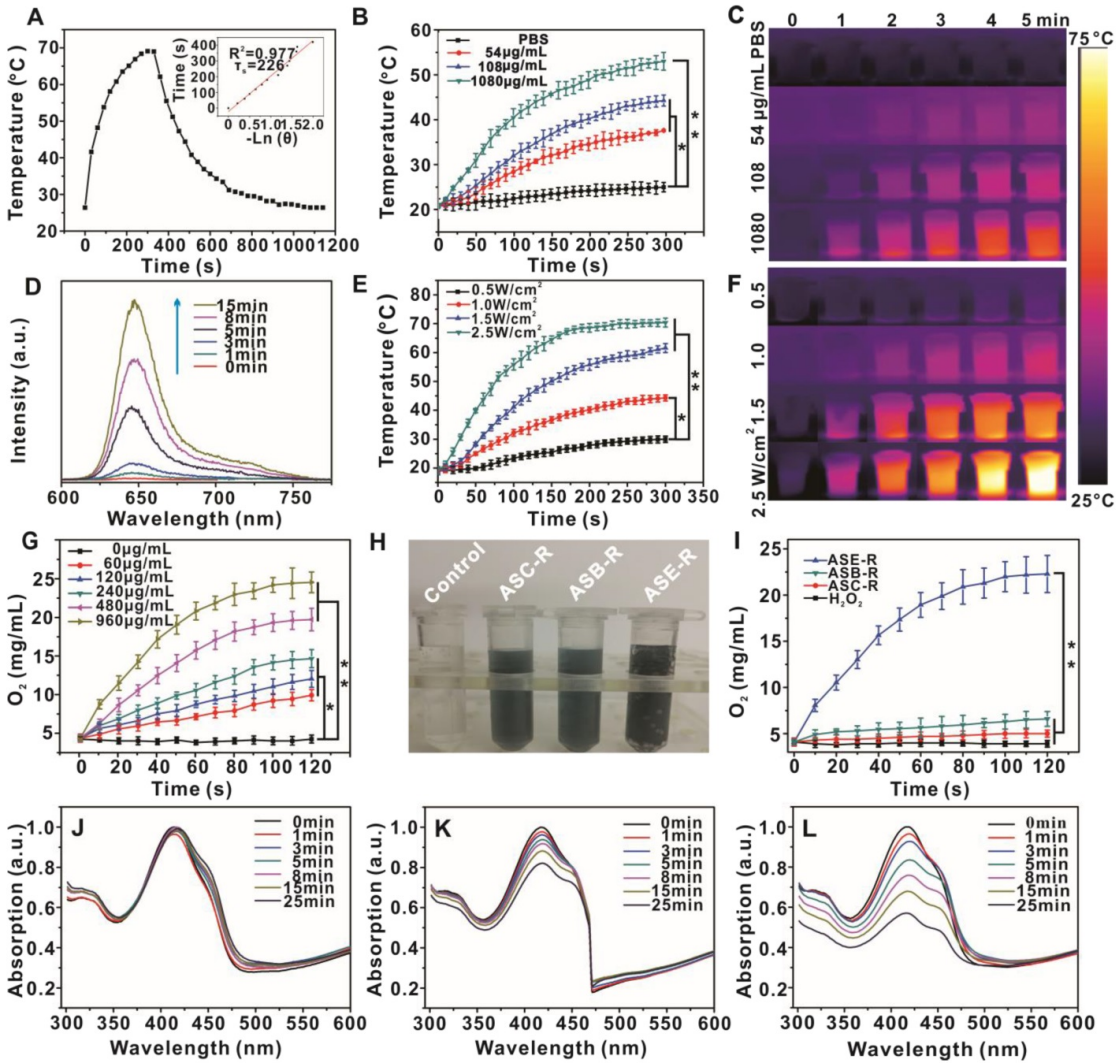
Temperature rise-drop diagram (A) and photothermal conversion efficiency diagram of ASCE-R (108 µg/mL) irradiated with 808 nm laser (2.5 W/cm^2^) for 5 min (insert); temperature rise curve (B) and thermal imaging (C) of ASCE-R irradiated with 808 nm laser (1.0 W/cm^2^); fluorescence spectra of the supernatant solution of ASCE-R irradiated with 808 nm laser (2 W/cm^2^) for different times (D); temperature curve (E) and thermal imaging of ASCE-R (108 µg/mL) irradiated with 808 nm laser with different power (F); oxygen production curve of different concentrations of ASCE-R in 1 mM H_2_O_2_ (G); oxygen production white light photograph (H) and oxygen production curve of different probes (I); absorption spectra of DPBF after irradiation of ASCE-R probe with 660 nm laser (J), 808 nm laser (K), 808 nm and 660 nm alternately (L), 808 nm: 1.0 W/cm^2^, 660 nm: 0.1 W/cm^2^.

ASCE-R also showed an excellent photothermal property identical to that of GNS (Figure 4A) according to the equation for the photothermal conversion efficiency: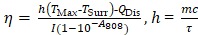, *τ* = 226 (Figure 4A, insert),* Q*_Dis_ = *h*×(*T*_Max water_ - *T*_Surr_), where *m* is the solution mass, *c* is the specific heat capacity of water, the laser power *I* is 600 mW, the absorption value *A*_808_ is 0.193 at 808 nm, *T*_Max_ is 69.9 ºC, *T*_Surr_ is 25.1 ºC, and *T*_max water_ is 33.8 ºC. The photothermal conversion efficiency of ASCE-R was calculated to be 28%, which exceeds the 23.7% conversion efficiency of gold nanorods 50. For the same laser intensity (1.0 W/cm^2^), the temperature of the system increased with increasing probe concentration (Figures 4B and 4C). When the probe concentration was 108 µg/mL and the laser power was 1.5 W/cm^2^, the temperature reached 60 ºC, which could result in cell death. For the same probe concentration, stronger irradiation power led to more significant temperature increases (Figures 4E and 4F). The probe was also characterized after irradiation and the results showed that the probe had the same morphology after irradiation when compared with the original probe, indicating good stability of the probe (Figure S4).These results demonstrated that ASCE-R possesses favorable photothermal conversion ability and can achieve both PTT and drug release.

Catalase immobilized on the surface of the probe can catalyze the conversion of H_2_O_2_ to oxygen 51 which can improve the photodynamic therapeutic effect. Therefore, the oxygen production capacity of the probe was investigated. Our results showed that with increasing ASCE-R concentration and time, oxygen increased gradually in solution reaching 23 mg/mL within 2 min at a concentration of 960 µg/mL ASCE-R. In contrast, the oxygen concentration remained low in the system without probe (Figure 4G) indicating strong catalytic oxygen production capacity of the probe. To confirm the source of oxygen, we used BSA (B) instead of catalase to prepare the ASB-R probe and found no significant change within the span of 2 min in the oxygen concentration in blank H_2_O_2_, ASB-R, and ASC-R without the enzyme-linked probe system.

However, the ASE-R probe showed clear bubble formation (Figure 4H), indicative of oxygen production which reached a high of 23 mg/mL (Figure 4I), confirming that the catalase in the probe could catalyze H_2_O_2_ to oxygen. The amount of encapsulated catalase was determined using the bicinchoninic acid protein assay, which is a detergent-compatible formulation for the colorimetric detection and quantitation of total protein 52. The probe ASE (Au@mSiO_2_@Catalase) was prepared and the loading efficiency of catalase in ASCE-R was calculated to be 9.6%.

In the ASCE-R probe, since GNS could quench the fluorescence of Ce6 in the mesoporous channels and inhibit the production of singlet oxygen, Ce6 was in an "off" state. When the probe released Ce6, it was converted to an "on" state as it was sufficiently distant from GNS. The molecule DPBF, a singlet oxygen detection reagent, was used to monitor the photodynamic behavior of the probe. The results showed that DPBF absorption at 415 nm did not change in response to probe irradiation with 660 nm laser for 25 min (Figure 4J) indicating no obvious singlet oxygen production. After irradiation with the 808 nm laser, DPBF absorption gradually decreased to about 20% within 25 min (Figure 4K), indicating the presence of singlet oxygen. When the solution was successively irradiated with 808 nm and 660 nm lasers for 25 min, the OD value decreased by about 50% (Figure 4L).

These results demonstrated that the probe produced considerable heat after irradiation with the 808 nm laser resulting in the breakdown of the phospholipid layer and the release of the encapsulated Ce6 from the mesoporous channels. This could be verified by the fluorescence caused by Ce6 release with prolonged irradiation time in the supernatant following centrifugation (Figure 4D). The fluorescence gradually recovered, indicating that the probe could quench Ce6 fluorescence to a large extent. When the probe was irradiated with the 808 nm laser, it could gradually release Ce6 which absorbed 808 nm light and produced a small amount of singlet oxygen. However, the efficiency of this conversion was low. Because the commonly used 660 nm laser in PDT could not produce the required photothermal effect at 0.1 W/cm^2^ (Figure S5), Ce6 did not leak from the channel to induce singlet oxygen production. When two types of lasers were used in alternating fashion, the photothermal effect using the 808 nm laser first released Ce6 from the channel, then irradiation with the 660 nm laser resulted in the production of a large amount of singlet oxygen. This demonstrated that the designed probe possessed an excellent “on-off” effect, and that the photosensitizer Ce6 was not leaked to produce singlet oxygen in the “off” state. However, once the particle was switched to the “on” state, a large number of singlet oxygen molecules were effectively produced under 660 nm laser irradiation, thus illustrating the intelligent design of our probe.

### Cytotoxicity and α_v_β_3_ receptor-mediated endocytosis of ASCE-R

**Figure 5:**
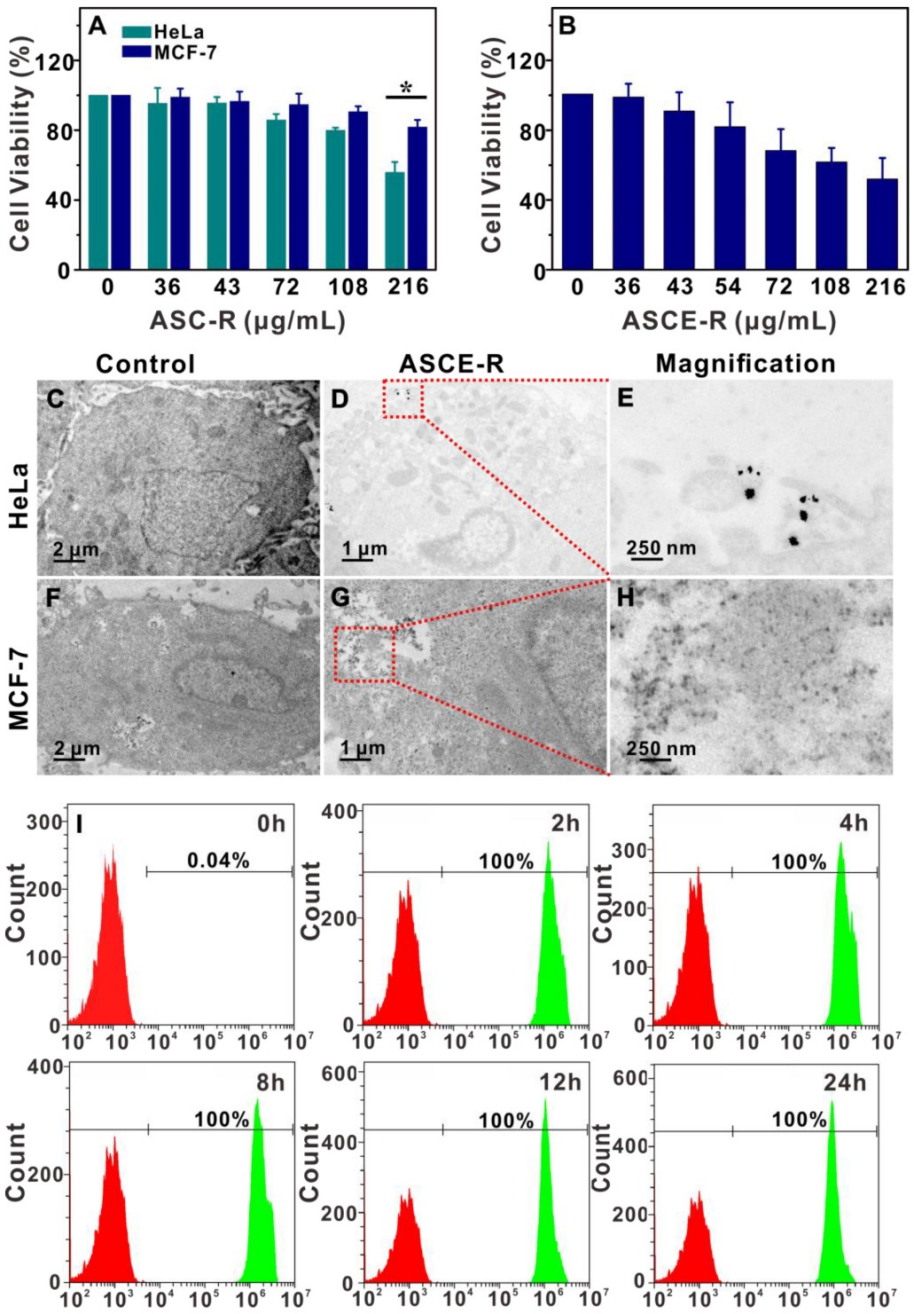
MTT results of HeLa and MCF-7 cells incubated with different concentrations of the ASC-R probe (A); survival rates of HeLa cells incubated with different concentrations of the ASCE-R probe (B); blank HeLa cells (C) and MCF-7 cells (F), and their uptake of ASCE-R probe by TEM (D, G) and local enlargement maps (E, H); flow cytometry quantitative fluorescence detection of HeLa cell incubated with the ASC-R probe at different times (I).

HeLa and MCF-7 cells were incubated with different concentrations of ASC-R probe without catalase for 24 h, and cytotoxicity of the probe was determined by the MTT assay. The probe at 216 µg/mL was more cytotoxic toward HeLa cells with the survival rate being 58.5% compared with ~87% in MCF-7 cells at the same concentration (p < 0.05) (Figure 5A). The lower survival rate of HeLa cells might be due to the high expression of the α_v_β_3_ receptors leading to specific targeting and increased internalization of the probe. When the toxicity of ASCE-R to HeLa cells was investigated by the MTT assay, the survival rate of HeLa cells was 52.0 % (Figure 5B) at the concentration of 216 µg/mL, which was slightly lower than in the presence of the same concentration of ASC-R. This may be due to the toxicity of the oxygen produced from H_2_O_2_ by catalase in the cells treated with ASCE-R 53.

Probe endocytosis was observed by TEM. Compared with untreated cells (Figure 5C), the probe (Figure 5D and E) could clearly be observed in HeLa cells that had been incubated with the ASCE-R probe. However, the probe was not detected in MCF-7 cells (Figure 5G and H) or untreated cells, where only a few cell fragments or impurities were observed (Figure 5F). These results indicated specific targeting effect of the ASCE-R probe on HeLa cells.

The uptake of the ASCE-R targeting probe by HeLa cells was also examined by flow cytometry. Compared with pre-incubation, almost 100% of tumor cells phagocytosed the probe 2 h after incubation. The fluorescence of Ce6 increased gradually with prolonged incubation time as the probe with excellent targeting ability was bound to cells and then endocytosed via a receptor-mediated pathway. The fluorescence of Ce6 in cells stabilized after 2 - 8 h (Figure 5I). It is possible that the phospholipid layer on the surface of the probe might be totally or partially removed after endocytosis slowly releasing the Ce6 into the cytoplasm to restore fluorescence even if the probe was not irradiated by a 808 nm laser.

### “On-off” state and catalase-enhanced PDT & PTT* in vitro*

**Figure 6:**
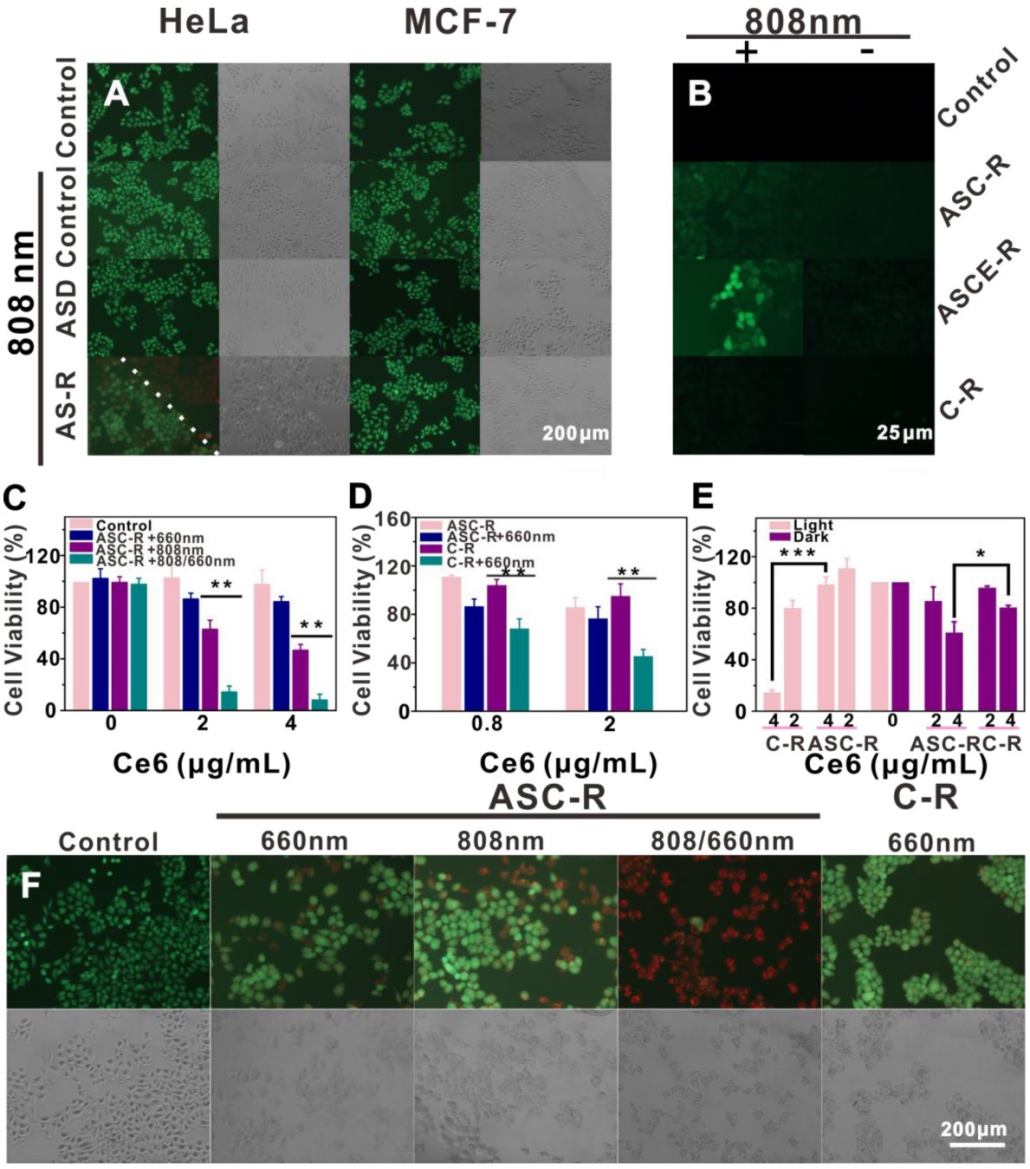
Fluorescence imaging of HeLa and MCF-7 cells stained with calcein-AM and PI after co-incubation with different probes and 808 nm laser irradiation (1 W/cm^2^) for 5 min or without irradiation, the white dotted line was the boundary of laser irradiation (A); fluorescence imaging of singlet oxygen produced by HeLa cells cultured with ASCE-R, ASC-R, and C-R after irradiation with 808 nm laser (1 W/cm^2^) for 10 min (B); MTT results of HeLa cells cultured with ASC-R irradiated at 808 nm (1 W/cm^2^) for 3 min, 660 nm (0.1 W/cm^2^) for 8 min and under alternate irradiation (C); MTT results of HeLa cells cultured with ASC-R,C-R at 660 nm (0.1 W/cm^2^) (D); MTT results of HeLa cells incubated with ASC-R and C-R under white light and in the dark, *: p<0.05, **: p<0.01 (E); fluorescence imaging after calcein-AM and PI staining HeLa cells incubated with ASC-R and C-R (1 µg/mL Ce6) after irradiation with 808 nm laser (1 W/cm^2^) for 3 min, 660 nm laser (0.1 W/cm^2^) for 8 min, under alternate irradiation (F).

To verify the targeted photothermal effect of the probe, AM and PI were used to stain living and dead cells, respectively, after various treatments. Both HeLa and MCF-7 cells in the blank group showed green fluorescence regardless of laser irradiation, indicating that laser irradiation alone could not cause cell death (Figure 6A). To investigate the photothermal effect of GNS alone, the AS-D probe, which was not loaded with Ce6, E, and the targeting peptide on the encapsulated phospholipid, was incubated with both Hela and MCF-7 cells. After laser irradiation, the cells still showed strong green fluorescence, indicating that the non-targeting probe AS-D could not kill cells as there was minimal cellular AS-D uptake that was insufficient to effectively increase the temperature of the tumor cells to kill them. When HeLa cells incubated with AS-R probe were laser irradiated, bright red fluorescence was observed in the irradiated area, while green fluorescence remained in the non-irradiated area. This indicated that HeLa cell apoptosis was due to the photothermal effect after irradiation once the AS-R probe was transported into the cells by the receptors. In control experiments, the irradiated area in MCF-7 cells with low receptor expression remained bright green, indicating that the low internalization of the probe was not sufficient to kill the cells after irradiation. Thus, our probe shows the desired targeting and photothermal effects.

Furthermore, a singlet oxygen kit was used to investigate the production of singlet oxygen via intracellular enzymatic catalysis in response to laser irradiation. To demonstrate the role of catalase, an 808 nm laser with weak photodynamic ability was used. The results showed a lack of singlet oxygen in the blank group regardless of laser irradiation (Figure 6B). After incubation of HeLa cells with ASC-R or ASCE-R for 4 h, green fluorescence in the cells under laser irradiation was significantly stronger than that in non-irradiated cells. Also, the fluorescence of ASCE-R was stronger than that of ASC-R due to the production of O_2_ from intracellular hydrogen peroxide genetated by catalase present in ASCE-R.

It has been reported that after 808 nm laser irradiation, photothermal damage to the phospholipid layer released hydrogen peroxide in the tumor cells, which when exposed to catalase 54 was converted to oxygen and water 55, 56. In contrast, the ASC-R probe had no catalytic ability for oxygen production; therefore, only a small amount of singlet oxygen was produced. The probe C-R did not produce heat under 808 nm laser irradiation and could not produce singlet oxygen even when a large amount of photosensitizer Ce6 was loaded. As previously reported, the photothermal effect and acidic environment of the tumor accelerated the destruction of the phospholipid layer 57 and the high concentration hydrogen peroxide in tumor penetrated the layer of R.

It is speculated that there are two mechanisms for the formation of singlet oxygen. First, the generated oxygen diffuses into the mesoporous silicon channel and receives light energy from the photosensitizer irradiation at 660 nm, producing in a large amount of singlet oxygen with high oxidation activity. Second, the electrostatic interaction between Ce6 and the amino group of the inner channel surface is weakened, leading to dissociation of Ce6 from the channel. After irradiation with the 660 nm laser, light energy absorbed by Ce6 is transmitted to oxygen to produce singlet oxygen.

After investigating the production of singlet oxygen by the probe, its PDT effect was examined. The Ce6 concentration was used to represent the concentration of the probe. The viability of cells cultured with the ASC-R probe decreased slightly after 660 nm laser irradiation while that of cells irradiated with the 808 nm laser diminished markedly demonstrating the effect of PTT (Figure 6C). However, when the cells were irradiated with the 808/660 nm lasers, cell viability decreased rapidly and there was a significant difference in the cell survival rate compared with laser irradiation with the 808 nm or the 660 nm laser alone (p < 0.01) at a Ce6 concentration of 2 µg/mL. As indicated, this might be due to the release of Ce6 under 808 nm laser and the activation of Ce6 by 660 nm laser releasing a large amount of singlet oxygen to kill the tumor cells. After incubation with the ASC-R probe alone, no significant differences were found in the cell survival rate regardless of whether the cells were irradiated with 660 nm laser, indicating that the photodynamic effect was not significant in this case. We speculated that most of the Ce6 was still in its "off" state in the channel (Figure 6D).

To verify this hypothesis, we synthesized the C-R probe. The survival rate of Hela cells treated with the C-R probe after irradiation with 660 nm laser was significantly different from that of pre-irradiated cells (p < 0.01), suggesting that Ce6 was in the "on" state after tumor cell target recognition. Therefore, a large amount of singlet oxygen was produced under 660 nm laser irradiation, which could kill the HeLa cell. This "on-off" effect of Ce6 in our probes was critical to avoid phototoxicity toward healthy cells.

Our experiments also showed that the cytotoxicity of ASC-R was lower than that of the C-R probe after 6 h of HeLa cells under white light. In particular, when Ce6 concentration was 4 µg/mL, the phototoxicity of C-R became evident as the survival rate of cells was only about 10%, while that of cells treated with the ASC-R probe exceeded 90%. These differences were statistically significant (p < 0.001), and implied that phototoxicity of Ce6 could be effectively avoided by our probe (Figure 6E). The survival rates of HeLa cells incubated with C-R (4 µg/mL) or ASC-R probe in the dark were also significantly different (p < 0.05). However, this difference was smaller than that under the white light and may be caused by the AS in ASC-R probe.

In summary, the probe released Ce6 under 808 nm laser irradiation and generated a large amount of singlet oxygen under 660 nm laser irradiation leading to remarkable cytotoxicity. Consequently, the effect was significantly better than that of PTT under 808 nm laser irradiation or PDT under 660 nm laser irradiation alone. To further verify this "on-off" effect of the probe, AM and PI were used to stain the cells after different treatments (Figure 6F). When HeLa cells were incubated with the ASC-R probe for 4 h and irradiated with 808 and 660 nm light, almost the entire irradiated area showed red fluorescence and extensive cell death due to hte combined effect of PTT and PDT. Green and red fluorescence coexisted when the cells were irradiated with 808 nm laser alone, indicating that more cancer cells died due to the photothermal effect. On the other hand, very little red fluorescence was observed when 660 nm laser was used alone and most of the cancer cells did not die. Similarly, weak red fluorescence was found when the C-R probe was irradiated using the 660 nm laser indicating a low level of tumor cell apoptosis. These results suggested that the photodynamic effect of the probe at low concentration (1 µg/mL Ce6) was not evident independent of the “on” or “off” state of Ce6 but the photothermal treatment effect was significant. However, PTT combined with PDT exhibited the best outcome, which fully reflected the "on-off" merit of the developed probe.

### “On-off” state and catalase-enhanced PDT & PTT* in vivo*

**Figure 7:**
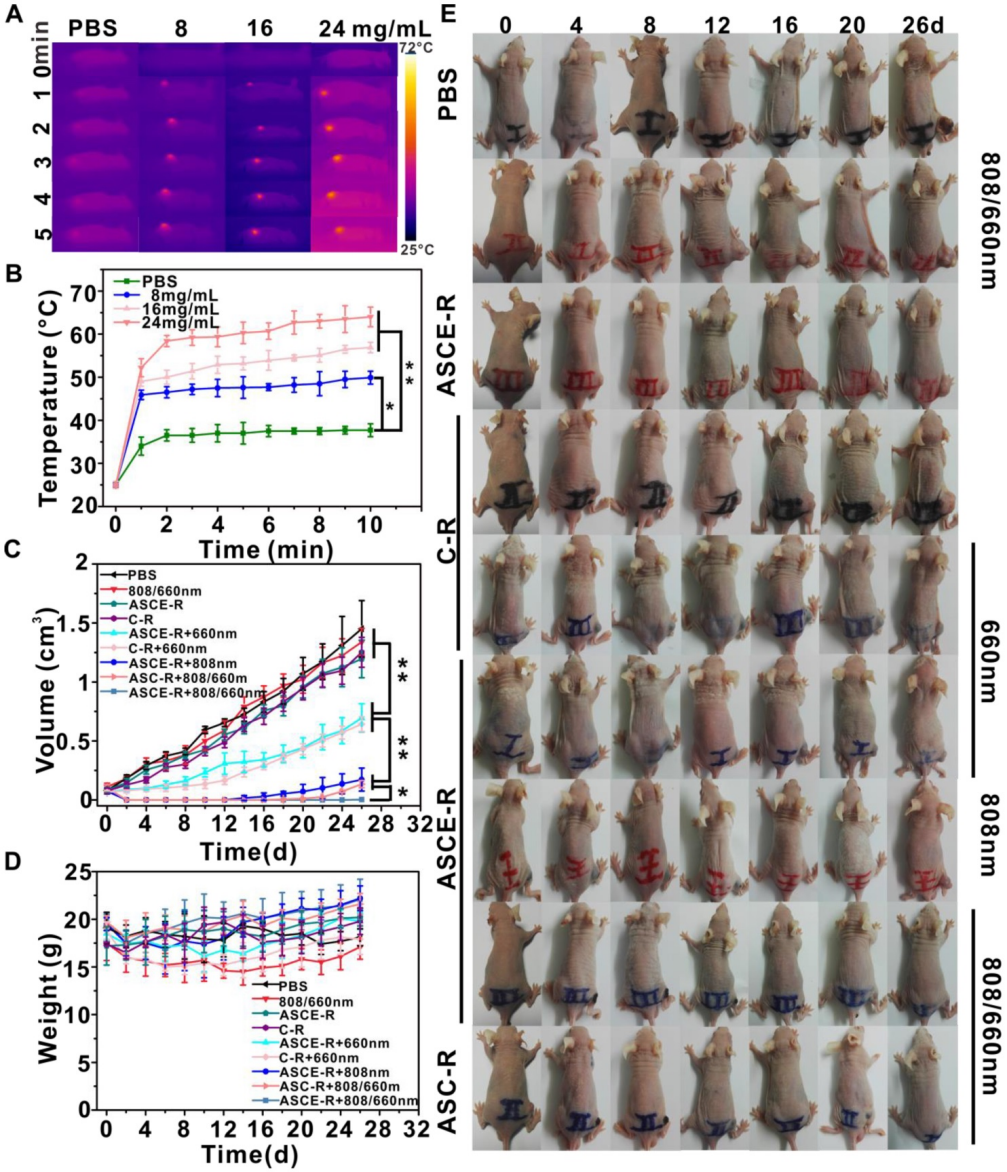
Thermography (A) and curve (B) of temperature change with time after PBS and ASCE-R were injected into tumor-bearing mice; tumor volume (C), body weight (D) and white light (E) after PBS, C-R, ASC-R, and ASCE-R probes were injected into tumor-bearing mice. (n=5*: p<0.05, **: p<0.01).

Next, the therapeutic effect of the probe was investigated on HeLa tumors *in vivo*. Tumor-bearing nude mice were injected with probes at different concentrations and were irradiated with 808 nm laser. The temperature of the control group (PBS treated) increased from 10 °C to 35 °C within 10 min, while in the probe-treated group, the temperature was higher than that of the control group during the same time. A probe concentration of 8 mg/mL increased the temperature of the tumor site to 45 °C (Figure 7A and B), which was sufficient to kill the tumor cells. After various probes were injected for different treatments, the size of the tumor was determined to evaluate the achieved therapeutic effect (Figure 7C). We found that the treatment effect could be divided into four categories:

- Changes in the tumors treated in the PBS, C-R, ASCE-R, and 808/660 nm alternating laser irradiation groups were similar and the tumor volume increased with time, indicating that C-R and ASCE-R had no significant therapeutic effect without laser irradiation, and that laser irradiation alone was futile.
- In C-R and ASCE-R groups irradiated with 660 nm laser, the sizes of the tumors were similar. However, tumor growth was slower and tumor volumes were less than those found in category I, and categories I and II showed a significant statistical difference (p < 0.01). For the C-R probe group, this may be caused by the toxic singlet oxygen produced by Ce6 in response to 660 nm laser irradiation after the probe entered the tumor cells. For the ASCE-R group, some Ce6 leakage could take place after the probe entered the cells. Following irradiation, singlet oxygen generated in the cytoplasm could affect both the metabolism and the growth of cancer cells. At the same time, the surface-modified catalase converted the H_2_O_2_ in the tumor microenvironment to O_2_ and alleviated the problem of hypoxia, thereby enhancing the effect of PDT. However, the continued increase in tumor volume suggested that PDT alone could not inhibit tumor growth.
- In the ASCE-R group, the tumors were destroyed after 808 nm laser irradiation. However, four animals began to relapse after 14 d. Nevertheless, the tumor disappeared after treatment with alternating 808/660 nm laser irradiation in the ASC-R group. Three tumors began to recur after 22 d. The changes in tumor volume in both groups were similar. However, the tumor growth rate was slower than that in category II with a significant statistical difference (p < 0.01), indicating that PTT and DT were more effective for the treatment of tumors, but could not completely eliminate the tumors.

In the ASCE-R group with catalase, the effect of alternating 808/660 nm laser irradiation was most remarkable. After treatment, all tumors disappeared in five mice, and only one mouse showed sign of recurrence after 26 d. A significant statistical difference was found in tumor size when compared with category III (p < 0.05), indicating that the ASCE-R group achieved the best therapeutic effect after alternating 808/660 nm laser irradiation. This probe exerted the combined effect of PTT and controlled PDT, while the surface-modified catalase converted high concentrations of H_2_O_2_ in the tumors to O_2_, thus reducing H_2_O_2_ in the tumor microenvironment, alleviating the problem of hypoxia, and improving the effect of PDT. At the same time, the skin of mice in this group showed no signs of edema, and ulceration during treatment, indicating that the probe prevented the damage caused by phototoxicity (Figure 7E). Weight monitoring of mice showed that the mice of each group lost weight 2 d after the start of the treatment. This may be a side-effect of the anesthetics, which led mice to lose their appetite, but the weight of the mice began to increase after 4 d (Figure 7D). In summary, the therapeutic effect of alternating 808/660 nm laser irradiation was better than that of 808 nm or 660 nm laser irradiation alone. The probe containing an enzymatic component relieved the poor PDT effect caused by hypoxia. Thus, the synergistic effect of two kinds of lasers indicated that the probe has low phototoxicity, superior targeting ability, and high efficacy with excellent application prospects.

### *In vivo* biocompatibility and safety

**Figure 8:**
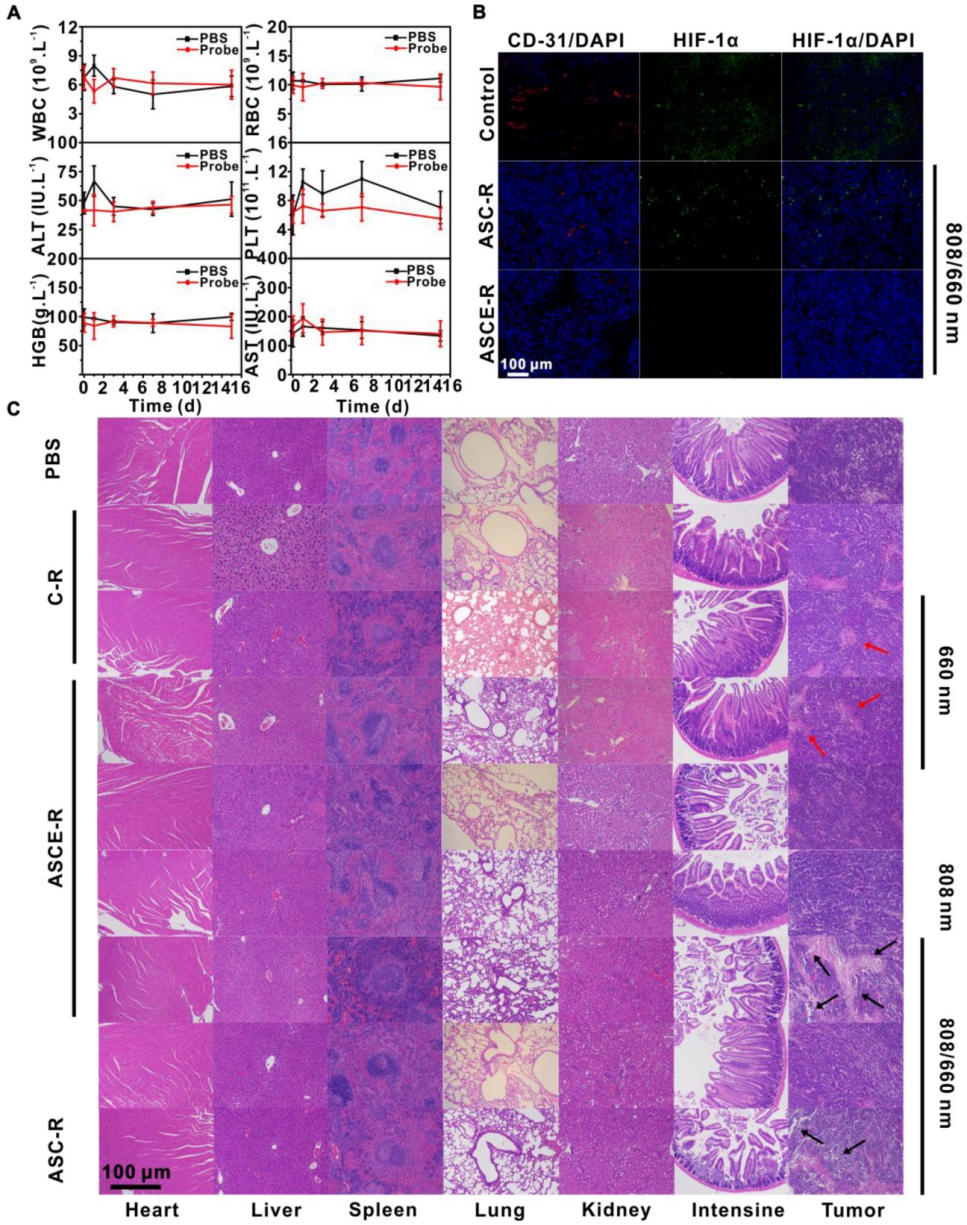
Blood analysis in normal mice injected with ASCE-R: ALT, AST, HGB, PLT, RBC, and WBC (A); DAPI ,HIF-1α antibody, and CD-31 antibody staining of tumors in HeLa tumor-bearing nude mice injected with PBS, ASC-R, and ASCE-R probes and irradiated with alternating 808 and 660 nm laser for 24 h (B); H&E staining of the heart, liver, spleen, lung, kidney, small intestine, and tumor in HeLa tumor-bearing nude mice injected with different probes and with different treatments (C).

To further verify the therapeutic effect of the probes on the tumor, H&E staining was performed on the treated tumor sections (Figure 8C). Our results showed that tumor cells in the PBS group and its laser-irradiated group, the ASCE-R group, and the C-R group grew normally with no obvious cell necrosis. Compared with the blank control group, the tumor cells in both C-R and ASCE-R groups irradiated with a 660 nm laser showed different degrees of cell necrosis, atrophy, and separation between the nucleus and the cytoplasm (red arrow). In both the ASC-R group irradiated with 808/660 nm lasers and the ASCE-R group irradiated with the 808 nm laser, the phenotypes were very severe. Especially, in the ASCE-R group subjected to alternating laser irradiation, a large area of necrosis (black arrow), significant separation between the nucleus and the cytoplasm, and severe destruction of cell structure were observed. This was consistent with the results obtained from tumor volume monitoring.

The localized hypoxia at the tumor site could induce the expression of hypoxia-inducible factor HIF-1α, thus enhancing the secretion of vascular endothelial growth factor (VEGF) and promoting angiogenesis and tumor development. To investigate the ability of the probe to alleviate localized hypoxia, both HIF-1α and CD-31 antibody staining (tumor vessel endothelial cell adhesion molecule) was performed 24 h after intratumoral injection with ASCE-R and ASC-R probes and alternating irradiation with 808 and 660 nm lasers (Figure 8B). The results showed that the green and red immunofluorescence of HIF-1α and CD-31 antibodies in the ASC-R group decreased compared with that of the control group, indicating that hypoxia in the tumor site was alleviated to a certain extent due to the PTT-promoted blood circulation at the tumor site. However, the presence of residual fluorescence implied that hypoxia was not effectively resolved. Neither strong green nor red immunofluorescence were observed in the ASCE-R group, demonstrating that tumor hypoxia was significantly relieved with ASCE-R administration due to the catalysis of H_2_O_2_ to produce O_2_, which effectively alleviated hypoxia and inhibited tumor angiogenesis.

Finally, the biocompatibility of the probe was tested *in vivo*. H&E staining of the heart, liver, spleen, lung, kidney, and the small intestine after different treatments showed clear cell outlines in each tissue, and the nuclear structures could be observed (Figure 8C). The lung showed alveolar ducts, bronchi, and other structures. No apparent abnormal lesions, such as cell necrosis, were found. These results indicated that organs from mice treated with the probe were not damaged compared with the control group. After injection of ASCE-R probe into normal mice, biochemical blood indexes and liver enzymes were measured (Figure 8A). The results showed that AST increased sharply 1 h after ASCE-R probe injection, indicating that the rejection of foreign substances by the liver was strengthened, and the AST concentration tended to be steady with prolonged time; no noticeable difference appeared compared with the control group at 15 d. The platelet count increased 1 d after probe injection, which was indicative of the normal organismal response to a foreign substance. Platelet count also stabilized after 15 d and the difference between the test and control groups was not significant. The change in red blood cells was similar to that of the control group at 15 d, which further indicated that the probe achieved good blood compatibility. The leukocyte count decreased significantly 6 h after probe injection; however, the difference between the probe group and the control group was not obvious, and the number of WBC tended to be steady and returned to normal. All these results indicated that the ASCE-R probe had only a small effect on blood and biochemical indexes of normal mice and therefore offered good biological safety.

## Conclusions

We designed an integrated novel theranostic probe that used GNS as its core and Ce6-loaded mSiO_2_ as its shell. After modification with catalase and c(RGDyK) as the targeting moiety that specifically recognizes tumor cells, we performed targeted PTT and PDT of tumors in tumor-bearing nude mice. Compared to other PS, the probe could actively and effectively target tumor cells, with minimal damage to healthy tissues. It was only when the probe reached the tumor site that it showed a "gated" release of PS through the photothermal effect of GNS. The probe was then activated and produced a large amount of singlet oxygen that was able to kill tumor cells and avoid early PS leakage during *in vivo* transport. At the same time, catalase immobilized on the surface of mSiO_2_ catalyzed H_2_O_2_ to produce O_2_ at the tumor site. This overcame the hypoxia-induced resistance of tumors to conventional PDT caused by the consumption of oxygen further improving the effectiveness of our PDT treatment. Our results showed a remarkable synergistic effect of PTT and catalase-assisted photocontrolled PDT achieving a significant cytotoxic effect on mouse tumors. In summary, this multi-functional, highly biocompatible probe shows broad prospects for various medical applications.

## Figures and Tables

**Table 1:** Synthetic materials and abbreviations of various probes

Probes	abbreviations
Gold Nanostars	GNS
Au@mSiO_2_	AS
Au@mSiO_2_-NH_2_	ASN
Au@mSiO_2_@Catalase	ASE
Au@mSiO_2_/Ce6	ASC
Au@mSiO_2_/Ce6@Catalase	ASCE
Au@mSiO_2_@DSPE-PEG	AS-D
Au@mSiO_2_@DSPE-PEG-RGD	AS-R
Au@mSiO_2_/Ce6@DSPE-PEG-RGD	ASC-R
Au@mSiO_2_@Catalase@DSPE-PEG-RGD	ASE-R
Au@mSiO_2_@BSA@DSPE-PEG-RGD	ASB-R
Au@mSiO_2_/Ce6@Catalase@DSPE-PEG	ASCE-D
Au@mSiO_2_/Ce6@Catalase@DSPE-PEG-RGD	ASCE-R
Ce6@DSPE-PEG-RGD	C-R

Note: Catalase is abbreviated as E; DSPE-PEG-RGD is abbreviated as R; DSPE-PEG is abbreviated as D; BSA is abbreviated as B.

## Abbreviations

GNS: Gold Nanostars
AS: Au@mSiO_2_
ASN: Au@mSiO_2_-NH_2_
ASC: Au@mSiO_2_/Ce6
ASCE: Au@mSiO_2_/Ce6@Catalase
AS-D: Au@mSiO_2_@DSPE-PEG
ASE: Au@mSiO_2_@Catalase
AS-R: Au@mSiO_2_@DSPE-PEG-RGD
ASC-R: Au@mSiO_2_/Ce6@DSPE-PEG-RGD
ASE-R: Au@mSiO_2_@Catalase@DSPE-PEG-RGD
C-R: Ce6@DSPE-PEG-RGD
ASB-R: Au@mSiO_2_@BSA@DSPE-PEG-RGD
E: Catalase
R: DSPE-PEG-RGD
D: DSPE-PEG
B: BSA
ASCE-D: Au@mSiO_2_/Ce6@Catalase@DSPE-PEG
ASCE-R: Au@mSiO_2_/Ce6@Catalase@DSPE-PEG-RGD
