# Supplementary material for: Intelligent gold nanostars for in vivo CT imaging and catalase-enhanced synergistic photodynamic & photothermal tumor therapy
Source: Theranostics. 2019 Jul 13;9(19):5424–42. doi: 10.7150/thno.33015 (PMC6735389; doi:10.7150/thno.33015)
Supplement: Supplementary file 1 — Supplementary figures. [file thnov09p5424s1.pdf]

Supporting Material

# Intelligent gold nanostars for *in vivo* CT imaging and catalase-enhanced synergistic photodynamic & photothermal tumor therapy

Lin Zhang[a], Xiao-Quan Yang[a,b], Jian-Shuang Wei[a], Xing Li[a], Huan Wang[c], Yuan-Di Zhao[a,b,]*

[a] Britton Chance Center for Biomedical Photonics at Wuhan National Laboratory for Optoelectronics–Hubei Bioinformatics & Molecular Imaging Key Laboratory, Collaborative Innovation Center for Biomedical Engineering, College of Life Science and Technology, Huazhong University of Science and Technology, Wuhan 430074, Hubei, P. R. China

[b] Key Laboratory of Biomedical Photonics (HUST), Ministry of Education, Huazhong University of Science and Technology, Wuhan 430074, Hubei, P. R. China

[c] Division of Endocrinology, Diabetes and Nutrition University of Maryland, School of Medicine Baltimore, MD 21201, USA

* Corresponding author.
Tel/Fax: +86-27-8779-2202. Email address: zydi@mail.hust.edu.cn (Y.D. Zhao).

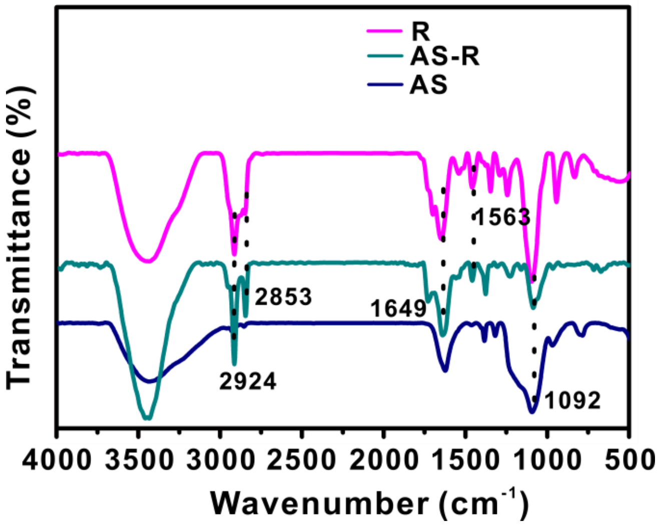


**Figure S1.** Infrared absorption spectra of AS-R, AS, and R.

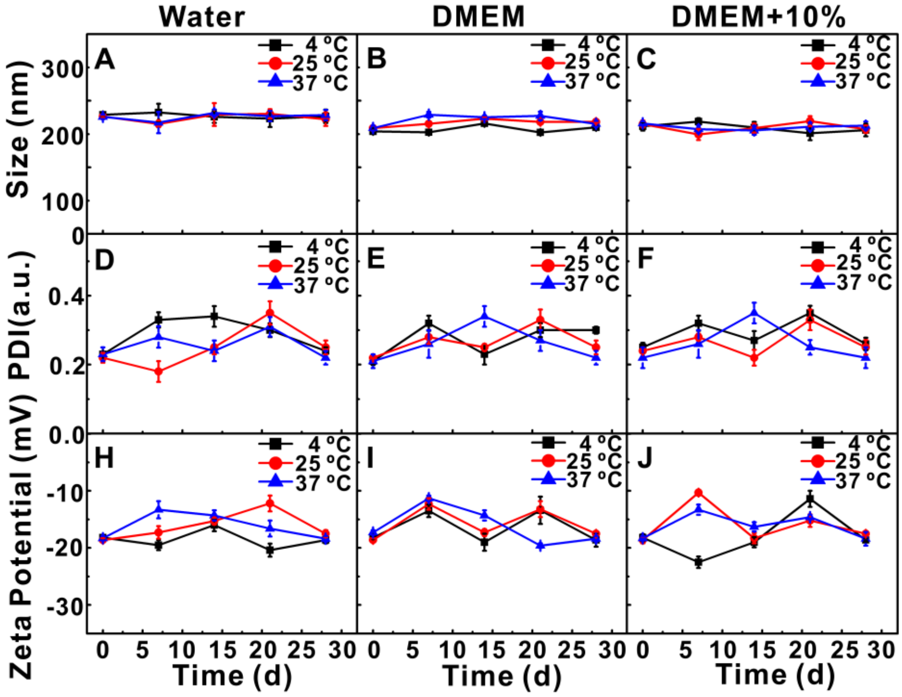


**Figure S2.** The particle size (A-C), dispersion PDI (D-F) and zeta potential of the ASCE-R probe in water, DMEM, and DMEM+10% serum changed with time (H-J).

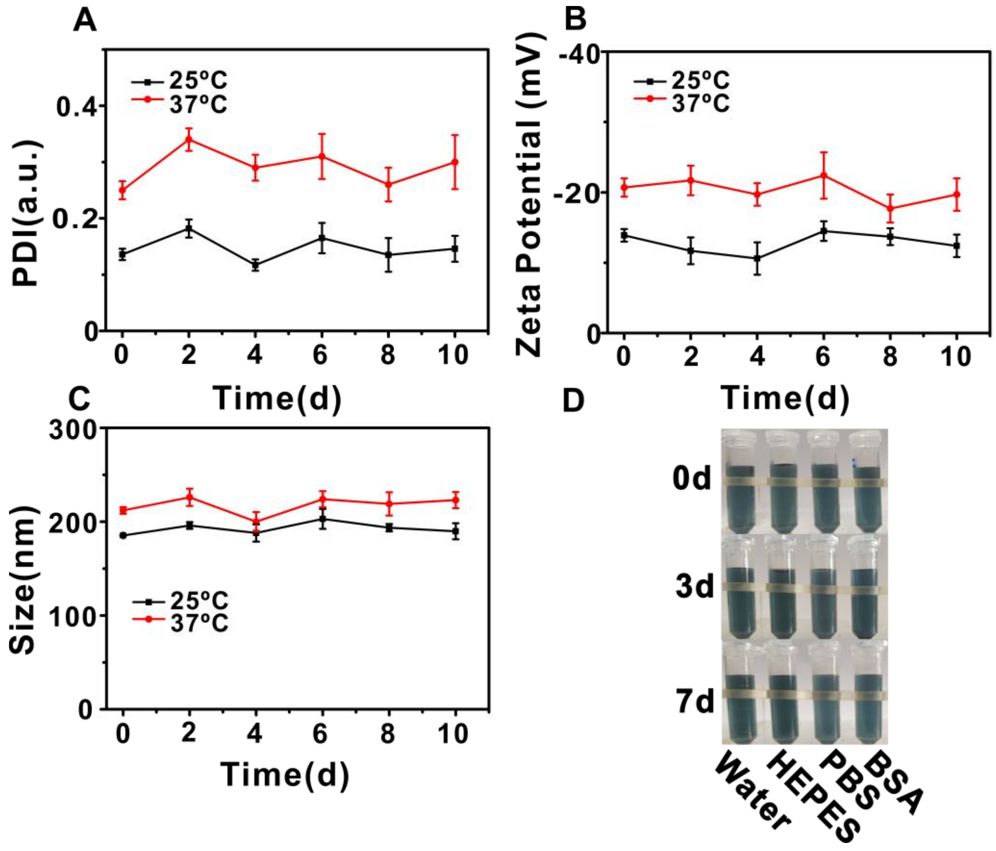


**Figure S3**. Changes in size (A), zeta potential (B) and PDI (C) of ASCE-R in PBS (pH=7.4) at 25 and 37 °C (n=3); digital photograph of ASCE-R in water, HEPES, PBS (pH=7.4), and bovine serum albumin at changed days (D).

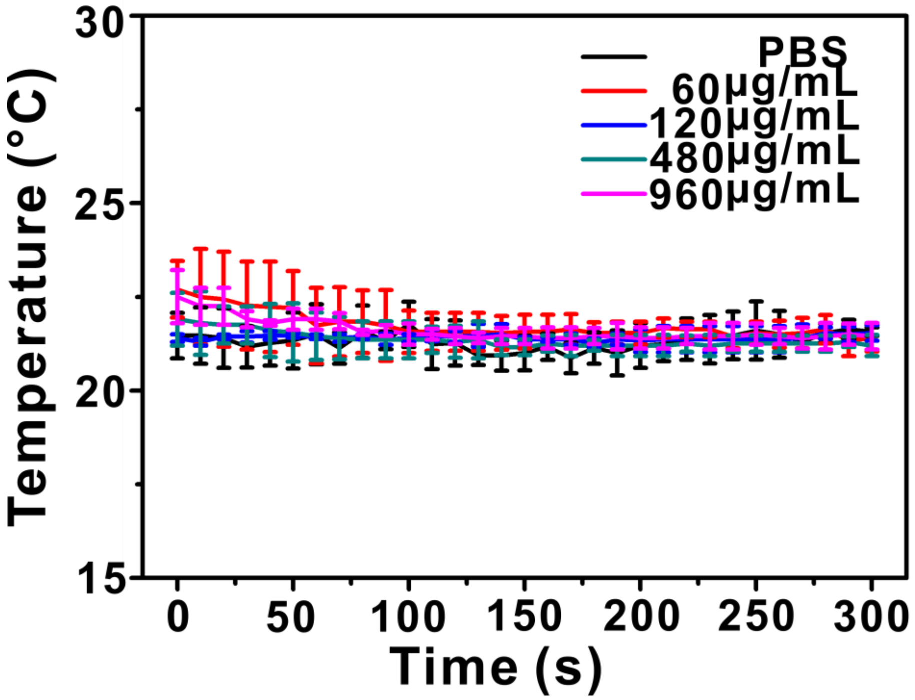


**Figure S4.** Temperature rise curves of ASCE-R with different concentrations irradiated by 660 nm laser (1.0 W/cm$^2$).

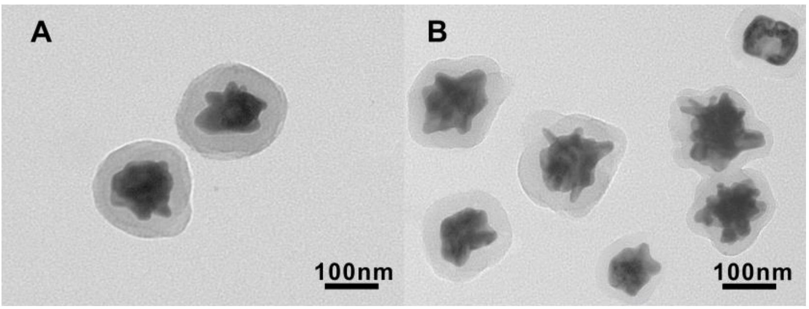


**Figure S5**. TEM of probe before (A) and after (B) irradiation at 808 nm laser (1 W/cm$^2$) for 3min, 660 nm laser (0.1 W/cm$^2$) for 8 min.
